# A Mobile App for Children With Asthma to Monitor Indoor Air Quality (AirBuddy): Development and Usability Study

**DOI:** 10.2196/37118

**Published:** 2022-05-23

**Authors:** Sunyoung Kim, Kaitlyn Stanton, Yunoh Park, Stephen Thomas

**Affiliations:** 1 Rutgers, The State University of New Jersey New Brunswick, NJ United States

**Keywords:** asthma, children, indoor air quality, mobile app, smartphone, mHealth

## Abstract

**Background:**

Indoor air quality is an important environmental factor that triggers and exacerbates asthma, the most common chronic disease in children. A mobile app to monitor indoor air quality could help occupants keep their indoor air quality clean. However, no app is available that allows children to monitor and improve their indoor air quality.

**Objective:**

Previously, we conducted a series of user-centered design studies to identify user needs and design requirements toward creating a mobile app that helps children with asthma to engage in monitoring and improving indoor air quality as part of their asthma management. Based on the findings from these studies, we created AirBuddy, a child-friendly app that visualizes air quality indoors and outdoors.

**Methods:**

This paper reports on the findings from a field deployment with 7 pediatric asthma patients, where we evaluated AirBuddy’s usability and usefulness in real-world settings by conducting weekly semistructured interviews for 8 weeks.

**Results:**

All participants positively responded to the usefulness and usability of AirBuddy, which we believe is thanks to the iterative, user-centered design approach that allowed us to identify and address potential usability issues early on and throughout the design process.

**Conclusions:**

This project contributes to the field of mHealth app design for children by demonstrating how a user-centered design process can lead to the development of digital devices that are more acceptable and relevant to target users’ needs.

## Introduction

Asthma is recognized as the most common chronic disease in children, affecting approximately 12% of children worldwide [1,2]. Its common symptoms include coughing, wheezing, chest tightness or pain, and difficulty breathing. Childhood asthma is especially concerning because it creates a substantial burden on the affected children and their families by requiring regular medical encounters, restricting the child's physical activities, and increasing the chance of school absences [3-6].

Asthma cannot be cured currently, but good management can control the disease and enable people with asthma to sustain a normal, active life. One important aspect of asthma management is avoiding or reducing environmental asthma triggers. Among the various triggers that contribute to excessive asthma morbidity, exposure to air pollutants is a significant environmental trigger that worsens symptoms and causes asthma attacks [7-10]. Since children spend most of their time indoors, the condition of the indoor air quality (IAQ) determines their exposures to many air pollutants [11,12]. Thus, it is crucial to keep IAQ clean and healthy for asthma management [13-15]. However, it is challenging to detect air pollutants with human perception because many air pollutants are invisible and thus impossible to detect with human senses. Furthermore, childhood asthma management is complex because pediatric patients with asthma rely on caregivers to manage asthma, and caregivers cannot fully keep track of the environmental triggers to which a child might be exposed [16]. A tool that allows children to monitor IAQ easily might help mitigate these problems by enabling them to reduce their exposure to air pollutants and make healthy choices themselves.

Mobile apps are increasingly available and used to facilitate various aspects of asthma management [17-20]. However, few apps are available for IAQ monitoring, and even fewer apps are designed for children’s use. Thus, this project aimed to create a mobile app for children with asthma to engage in monitoring and improving IAQ. Specifically, we aimed to adopt an iterative, user-centered design approach by involving potential users throughout the design process to employ their perspectives in design. Previously, we investigated children’s perspectives and design requirements through a review of existing applications and 2 sets of semistructured interviews with 12 children with asthma [21]. Based on the findings from these previous studies, we developed AirBuddy, a mobile app that visualizes air quality indoors and outdoors in a child-friendly manner. As the last stage of this project, this paper reports on the outcomes of a 2-month field deployment where we evaluated the usability of AirBuddy in real-world settings.

## Methods

### System Development

**Figure 1:**
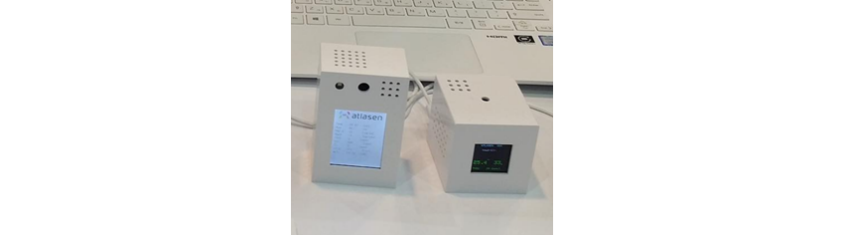
Atlasen Pico, an Air Quality Index monitoring station.

**Figure 2:**
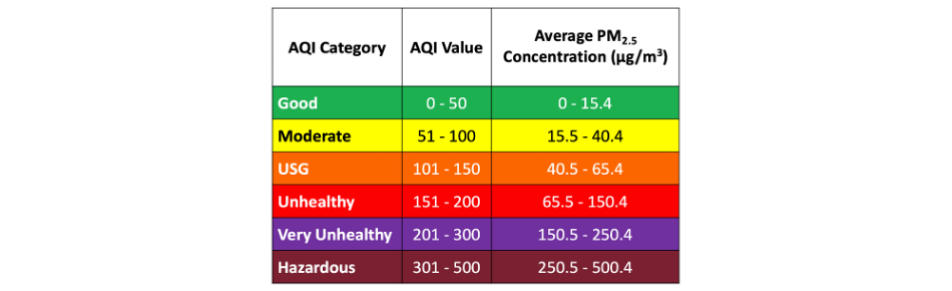
The Air Quality Index (AQI) category for fine particulate matter (PM_2.5_) [22]. USG: Unhealthy for Sensitive Group.

For IAQ sensing, we used an off-the-shelf sensor that continuously measures the levels of 5 air pollutants: fine particulate matter (PM_2.5_), carbon monoxide (CO), carbon dioxide (CO_2_), total volatile organic compounds (TVOC), and nitrogen dioxide (NO_2_) (see Figure 1). This sensor transmits the measurements of these air pollutants to the server every 15 seconds. Then, the system determines the current IAQ level based on the level of the air pollutant that has the lowest Air Quality Index (AQI) rating [22] among the five air pollutants. For instance, if the 5-minute average of PM_2.5_ is 20 μg/m^3^, and its AQI category is the lowest among the air pollutants (see Figure 2), the system determines the current IAQ level as “moderate.” The server sends the determined IAQ level to AirBuddy every 5 minutes, and the AirBuddy app displays IAQ as “moderate.” In addition, outdoor air quality data are retrieved from an AirNow API (application programming interface) [22] that provides current air quality data by zip code.

**Figure 3:**
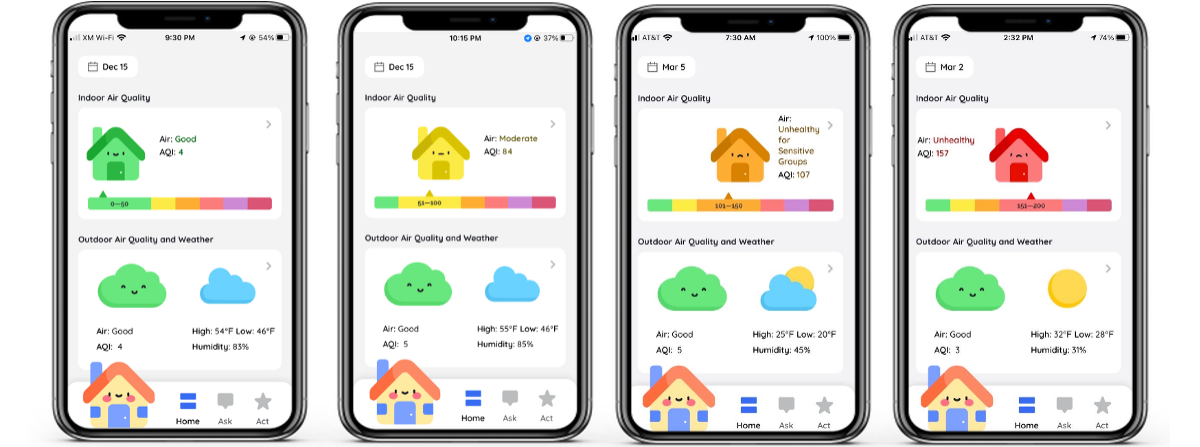
A final design of AirBuddy: home screen indicating different indoor air quality levels.

**Figure 4:**
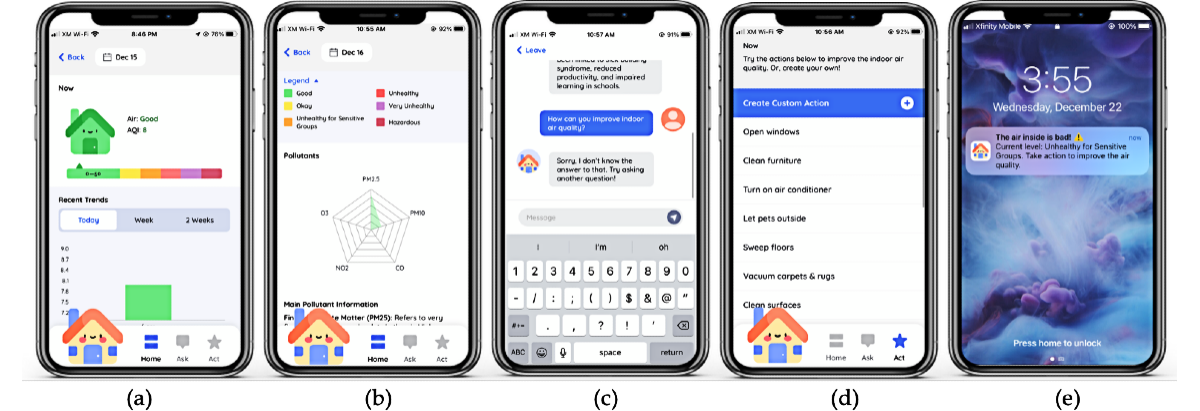
A final design of AirBuddy: (A) a bar graph for indoor air quality (IAQ) history; (B) a spider web for five air pollutants; (C) Airic, a chatbot; (d) a list of actions to improve IAQ; and (E) a push notification.

The AirBuddy app consists of a home page and three sub menus. At the top of the home page is the IAQ information pane with a house icon, an IAQ label with a numerical AQI value, and a horizontal AQI color strip to indicate the current IAQ level (see Figure 3). The color of a house icon and its location on a color strip change according to the current IAQ level based on an AQI color code [22] (eg, green for good IAQ, yellow for moderate IAQ, and red for unhealthy IAQ, see Figure 2). Below that is an outdoor information pane with a cloud icon for outdoor air quality and a weather icon. We juxtaposed these two icons to convey outdoor air quality information as part of outdoor weather information. When a user clicks anywhere in the IAQ information pane, the app moves to an IAQ detail page where a chart of recent IAQ trends is displayed (see Figure 4A).

Further below in this screen, we presented more details about color codes with legend labels and a spider web for five air pollutants (see Figure 4B). Next, clicking the house icon at the bottom left corner of a navigation bar brings up a chatting page where a user can interact both verbally and via typing with Airic, a chatbot, to ask any question relating to IAQ and asthma management (see Figure 4C). In addition, we provided a list of recommended actions that the user can take to improve the IAQ (see Figure 4D). Lastly, we implemented a push notification to notify a user when IAQ gets worse than the “good” range (see Figure 4E).

### Participant Recruitment

Children aged 8 to 12 years with asthma, as determined by the Guidelines for the Diagnosis and Management of Asthma [23], were eligible to participate in this study. We determined this age range for the participants because a child at the age of 8 years starts to understand abstract terms and complex sentences, develops the ability to read and critically analyze what they read, and shifts from learning to read to reading to learn [24]. Thus, children in this age range can use digital devices with complex and abstract languages for autonomous tasks [24]. Children were not eligible if they could not read or speak English, did not own a smartphone or other equivalent devices, or their involvement was deemed inappropriate by the pediatrician concerning their mental and physical conditions.

We recruited all participants from the pediatric pulmonology department of a tertiary hospital in an urban area. We first described the purpose of the study to a pediatrician, and the pediatrician shared us with a list of eligible patients upon their caregivers’ approval. Then, we approached by telephone caregivers of the patients to ask for their interest in participating in the study. A total of 7 participants were recruited (5 females and 2 males; mean age 10, SD 1.9 years; see Table 1). All caregivers and their children provided consent electronically prior to participating in the study.

**Table 1:** Participant demographics.

Participant ID	Age (years)	Gender	Asthma severity
P1	12	Female	Moderate
P2	8	Male	Moderate
P3	9	Female	Intermittent
P4	10	Female	Moderate
P5	10	Female	Intermittent
P6	8	Female	Intermittent
P7	12	Male	Mild

### Data Collection

We conducted a field deployment for 8 weeks. During the study, we conducted weekly interviews with each participant (8 interviews per participant in total) to investigate pediatric patients’ use of AirBuddy in their everyday lives over time.

**Figure 5:**
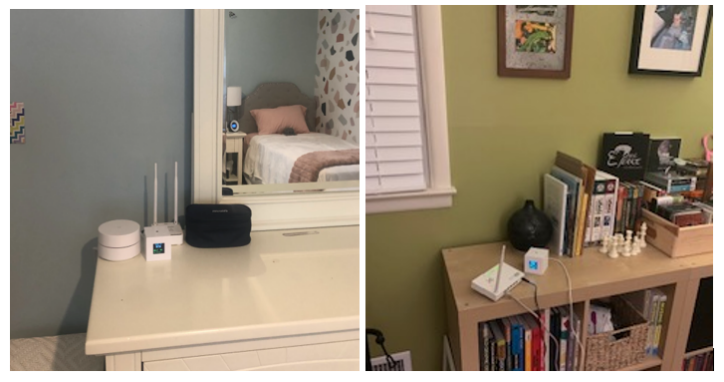
The location of an indoor air quality sensing unit for a deployment study: a vanity in a child’s bedroom (left) and a bookshelf in a living room (right).

Before initiating the study, we visited each participant’s home to set up an IAQ sensor in the room of their preference (eg, a child’s bedroom or living room; see Figure 5) and install AirBuddy on their smartphone. After setup, we described how the IAQ sensor works to the participants and their parents and gave basic instructions on how to use AirBuddy, which we introduced as “a mobile app that allows you to monitor air quality in your home and outside and find information about actions to take for asthma management.” Then, we conducted the first interview. Additionally, parents filled out a survey to inform us of the participants’ basic demographic information, including age, gender, and asthma severity. Lastly, participants were told to freely use AirBuddy as much as they wanted throughout the study period. They were given the contact information of the research team if they needed technical support.

The interviews focused on the following four topics: (1) how children initially perceive and respond to AirBuddy, (2) how they use it in their daily lives, (3) what motivates or prevents their use of the device, and (4) how their engagement in IAQ changes over time. Accordingly, we constructed three sets of open-ended interview questions to explore these topics in three phases. The first phase focused on understanding the general perspectives about IAQ and initial impressions of AirBuddy in the first interview. The second phase focused on exploring the user experience in-depth, including patterns of AirBuddy use, engagement in IAQ, and factors contributing to or preventing children’s engagement in monitoring IAQ throughout the deployment duration, except for the last interview. Finally, the third phase focused on exploring suggestions for improving AirBuddy and reviewing the overall reflection on the app in the last interview. Each interview lasted up to 60 minutes. After the study was complete, the research team visited the participants’ homes to pick up the IAQ sensor. All participants were compensated with a US $80 e-gift card.

All interviews except the first interview were conducted virtually using videoconferencing software of the participants’ choice (eg, Skype, Zoom, Google Duo). While all interview questions were asked to the children, we encouraged their parents to join the interview and share their thoughts if they wanted.

### Ethics Approval

This study was reviewed and approved by the Rutgers University institutional review board (reference #:Pro2019000875) prior to the conduct of the research.

### Data Analysis

We analyzed all interview data using thematic analysis to have significant patterns and themes emerge across data sets through the Grounded Theory process of open coding, axial coding, and selective coding [25]. First, we conducted open coding to identify and code concepts significant in the data as abstract representations of events, objects, happenings, actions, etc. Then, we grouped the related concepts created by open coding into a category to demonstrate conceptual phenomena using axial coding. Phenomena refer to repeated patterns of events, happenings, actions, and interactions that represent people’s responses to problems and situations. Finally, we followed the selective coding process to integrate all conceptual phenomena extracted from axial coding into a single storyline through building relationships across phenomena.

## Results

### Responses to the Graphical Interface

Initial impressions of AirBuddy’s graphical interface were positive across all participants. In the first couple of interviews, all participants expressed overall contentment with the aesthetics of the interface and positively mentioned its graphical components, including the vivid colors, color-coded icons, and child-friendly emojis.

The little characters are really cute. I like the house with the question marks and frowny faces or confused faces. What I like to do mostly with the app is that I can check the air numbers, good or bad. I like to do that because most people can't. [Participant P3, week 1]

I like the colors that are bright and popped. And it’s easy to understand the color of the house and air quality. Red means bad, yellow means okay, and green means good. The house is green now. It says that the air is good inside. [Participant P5, week 2]

As the usage continued, the interfaces presenting detailed information about IAQ (see Figure 4A,B) were perceived differently by different participants. Even though the age range of our participants was not wide (school-age children 8-12 years old), participants in different age groups expressed different reactions. Most of the older participants (aged 10 years and older) were content with and easily comprehended the detailed information on IAQ early in the study. However, younger participants (aged 9 years and under) expressed difficulty interpreting the presented information and complained about its complexity, especially that of the bar graph and a spider web. This suggests the need for more tailored considerations of effectively conveying information to children in different developmental stages.

I think everything is pretty self-explanatory. Something that I like is the graphs and how it tells you what you need to know. It doesn't go into all these like super technical things that not every kid will understand. It just gets to the point. [Participant P1 W1]

I am a little confused about the spider graph thing because I’m not good with those. I don't have a clue what it means. I’m fine with the bar graphs though. [Participant P6, week 2]

Nevertheless, even younger participants gradually learned to interpret IAQ information presented in AirBuddy as they continued using it over time. We provided instructions on how to interpret IAQ information whenever needed to those who had difficulty understanding it, but support needs significantly decreased after several weeks of app use. In all, we confirmed that AirBuddy is easy for children to use.

AirBuddy is a very good and informational app that teaches you about air quality around you and how to deal with it. It gives me good resources. At first, it was a little confusing because there were so many stuff. But now it is easy to navigate. [Participant P6, week 4]

### Motivations for App Use

One of the key questions we sought to investigate was what motivates or prevents children’s use of AirBuddy. To explore this, we asked when or why they opened AirBuddy to check air quality in every interview. Two prevalent answers emerged as triggers for app use. The most prevalent motivation to use AirBuddy was to inquire into the cause of their asthma worsening. Throughout the study, we had numerous statements about participants using AirBuddy to check the air quality indoors and outdoors whenever they had difficulty breathing. In most cases, participants found the air quality in the surrounding area worsened when they checked upon the occurrence of an asthma symptom. Consequently, participants learned that worsening air quality significantly contributes to asthma exacerbation. This demonstrates that AirBuddy is an effective tool for pediatric patients to check and confirm the source of an environmental asthma trigger.

I check the app when I'm not feeling too good or when I’m wheezing. Last week when I couldn’t breathe well, I opened AirBuddy to check if the air quality was okay, bad, or unhealthy. It was yellow. I think a person that has asthma would love it as it tells you how the air around you is. [Participant P4, week 4]

On Sunday, I felt my throat was itchy and I was struggling for air, and that's when I quickly checked AirBuddy. It said air quality is unhealthy and I told my mom. And she turned on the AC and opened the windows. [Participant P5, week 5]

An equally prevalent trigger was the app’s push notification to notify a user when the IAQ worsens. Once participants learned a significant relationship between air pollution and asthma worsening, they considered IAQ worsening seriously and took prompt action as soon as they received a push notification.

I’ve gotten notifications from the app when my air quality was bad. It’s like telling me “Hi [username], the air quality outside is bad you better stay inside.” A reminder helps a lot. [Participant P6, week 6]

Apart from these two triggers, participants frequently checked IAQ out of curiosity in the first couple of weeks. As their app use continued, however, this use pattern significantly decreased, which we believe was due to the novelty effect, and the participants relied solely on the two aforementioned triggers for their AirBuddy use. Only a few participants developed a routine to check IAQ over time.

Yesterday, we were looking at the graph. And it was funny because we noticed the graph was yellow at 6 AM and then went back to green at 8 AM. So, mom and I were trying to figure out what caused that. I think it’s heat. [Participant P1, week 2]

I only think about air quality when I'm having trouble breathing. So, if I'm fine, then it doesn't really faze me. [Participant P5, week 6]

Consequently, many participants reported that the frequency of app use gradually decreased as the study proceeded. They pointed out the lack of interactivity and fun aspects in AirBuddy as the main reason for their decreased app use. Accordingly, most participants suggested ideas to improve the app, related to adding more interactivity and fun features.

I’m completely comfortable using it. But I forget to use it because it is a little boring without much interaction. In fact, looking at air quality's not that fun. My favorite is watching a streaming site or FaceTime. [Participant P2, week 7]

“It would be cool if I could compare my data to other people's air quality. If people could see that you had bad air quality, they might want to go over to your house to fix your air quality. Or you could have a setting where you would talk to each other about low-quality air day or how you felt that day. [Participant P7, week 8]

### Engagement in Indoor Air Quality

Regardless of the app use frequency, we found that the participants’ overall experiences with AirBuddy remained positive from start to finish. All participants confirmed that AirBuddy helped promote their engagement with IAQ and increase their awareness and understanding of the effect of air quality on asthma. We received commending statements about the usefulness of AirBuddy in managing asthma and improving IAQ from every participant in the final interview. In particular, they found it helpful to acquire IAQ information whenever needed that was critically related to their asthma conditions but could not be obtained otherwise. In all, the study confirmed that AirBuddy is beneficial for children with asthma to effectively engage in and monitor IAQ information to manage their asthma conditions.

The app made me think about air quality a little more and more aware of my asthma. I never really put any thought into it before. Now, when I got the notifications of bad air quality, even if I am not at home, I think about how my breathing is…The other day when I was at play practice, I got a notification saying that my house had bad air quality. So, I texted and asked mom what she was doing and then thought about my breathing. [Participant P2, week 8]

It's awesome that I get to see how the air is around me, like whether I should turn on the AC or do anything to help my breathing. Before I was introduced to the app, I never thought about air quality. I've been doing the daily medicine a good three months, but it's just daily thing and I didn’t think about it. But now I got AirBuddy, and it makes me think about air quality a lot more and makes me more aware of keeping air clean and healthy. [Participant P7, week 8]

## Discussion

### Principal Findings and Study Strengths

This project aimed to create AirBuddy, a mobile app for children with asthma to monitor IAQ. To achieve the goal, we employed a user-centered design process whereby designers focus on the users and their needs in each phase of the design process by putting users at the center of product design and development [26]. Previously, we conducted a series of design studies, including reviewing existing systems, brainstorming ideas, wireframing, and high-fidelity prototyping, through which we iteratively revised and improved the app to assure that children can use it efficiently, effectively, and reliably [21]. Building on the previous work, this paper reports on a system development and its 2-month field deployment, the last step of a user-centered design process, in which we investigated how potential users would use AirBuddy in real-world settings. Overall, all participants positively responded to the usefulness and usability of AirBuddy, which we believe is thanks to the iterative, user-centered design approach that allowed us to identify and address potential usability issues early on and throughout the design process.

### Supporting Timely Access to Needed Information for Sustained Engagement

Technologies are considered successful not only when they are usable but also engage users [27], as the increased engagement has proven to be positively associated with solving problems [28]. In the past few decades, the health-informatics and human-computer interaction communities have recognized the importance of understanding and designing to promote engaging experiences with mHealth tools for effective health management [29-31]. Sustained engagement with mHealth tools has been proven to motivate patients to better adhere to health interventions and positively impact health behaviors and clinical outcomes [32,33]. Therefore, researchers have sought to determine elements for promoting effective and sustained engagement with mHealth tools [34,35]. Widely used design elements to boost sustained engagement include reminders, reward systems, and gamification [36,37].

One of our key design considerations was to ensure children’s sustained engagement with AirBuddy and thus sustain them to monitor IAQ over time. To serve this goal, we first considered implementing gamification features in the app design in the previous work [21], which has been widely employed to keep users, particularly children, hooked to the system [38,39]. However, we eventually discarded the idea for the following reasons. First, the IAQ in modern buildings in urban areas mostly stays healthy, except when indoor activities that negatively affect IAQ occur (eg, cooking, cleaning, increased humidity and temperature, or inadequate ventilation [40]) or when outdoor air pollutants penetrate indoors [41]. Thus, we concluded that it is more important for occupants to catch the moment of IAQ worsening and take prompt action to remove air pollutants rather than constantly checking the state of the IAQ. Second, we were concerned about providing children with yet another game app among the plethora of online games. While educational games have proven to be great tools for children to learn and drill specific skills [42,43], there is also a growing concern about increased screen time in children and its potentially adverse effects on health [44]. In the end, the crux of IAQ monitoring is on identifying the right moment to take proper action rather than the practice of monitoring itself. Thus, instead of aiming to keep a user’s attention constant in monitoring IAQ, we focused on drawing the user’s attention only at the point of interest—IAQ worsening. To that end, we implemented a push notification feature to alert users when IAQ worsens.

Participants expressed a feeling of boredom as their app use continued and pointed out the lack of fun features in AirBuddy as its primary area for improvement. Despite this drawback, all participants positively valued AirBuddy. While AirBuddy did not provide any entertaining features to keep users attached to the app, the participants concurred with its utility, in that the app promoted them to be more aware of managing their asthma condition and keep the IAQ clean and healthy. In the end, the ideal situation is one in which IAQ always stays good, so occupants do not need to pay attention to it. Thus, the onset of users’ asthma symptoms and push notification when IAQ worsens were sufficient in sustaining users’ engagement with the app without increasing their screen time. This finding implies that designing for children’s engagement can be realized not only by keeping them attached to the system—gamification—but also by enabling timely access to needed information. The next step is to explore ways to make available needed information for the users in a timely and relevant yet entertaining manner to boost sustained engagement with mHealth apps.

### Limitations

Our findings must be evaluated under the consideration of limitations. First, our sample size was small (N=7), and thus our participant pool may not represent a general children population. We initially planned to recruit more participants but were unable to due to the COVID-19 pandemic. Because people with asthma are particularly vulnerable to viral respiratory tract infections, most parents of pediatric patients with asthma were reluctant to participate in this study, which included 2 home visits. After recruiting participants for over 9 months, we decided to stop recruiting and proceed with the study with 7 participants. Second, we used convenience sampling for recruitment by recruiting participants from a children’s hospital in an urban area, which also runs the risk of compromising generalizability. Selection bias or unmeasured factors (eg, the homogeneity of participant characteristics and socioeconomic status) might have influenced the responses.

### Conclusion

This project designed, developed, and evaluated AirBuddy, a mobile app for children with asthma to monitor IAQ through a user-centered design process. This paper contributes to the field of mHealth app design for children by demonstrating how a user-centered design process can lead to the development of digital devices that are more acceptable and relevant to target users’ needs. A similar user-centered design approach can be effectively applied when designing mHealth apps for children to address self-management needs for other pediatric conditions.

## Abbreviations

AQI: Air Quality Index
IAQ: indoor air quality
PM_2.5_: fine particulate matter
TVOC: total volatile organic compounds

## References

[1] Mazurek JM, Syamlal G (2018). Prevalence of asthma, asthma attacks, and emergency department visits for asthma among working adults — national health interview survey, 2011–2016. MMWR Morb. Mortal. Wkly. Rep.

[2] CDC (2018). gov. CDC - asthma - data and surveillance - asthma surveillance data.

[3] Borhani Fariba, Najafi Maral Kargar, Rabori Eshaq Dortaj, Sabzevari Sakkineh (2011). The effect of family-centered empowerment model on quality of life of school-aged children with thalassemia major. Iran J Nurs Midwifery Res.

[4] Diette GB, Markson L, Skinner EA, Nguyen TTH, Algatt-Bergstrom P, Wu AW (2000). Nocturnal asthma in children affects school attendance, school performance, and parents' work attendance. Arch Pediatr Adolesc Med.

[5] Milton B, Whitehead M, Holland P, Hamilton V (2004). The social and economic consequences of childhood asthma across the lifecourse: a systematic review. Child Care Health Dev.

[6] Erbas B, Kelly A, Physick B, Code C, Edwards M (2005). Air pollution and childhood asthma emergency hospital admissions: Estimating intra-city regional variations. International Journal of Environmental Health Research.

[7] D'Amato G, Liccardi G, D'Amato M, Holgate S (2005). Environmental risk factors and allergic bronchial asthma. Clin Exp Allergy.

[8] Clark NA, Demers PA, Karr CJ, Koehoorn M, Lencar C, Tamburic L, Brauer M (2010). Effect of early life exposure to air pollution on development of childhood asthma. Environmental Health Perspectives.

[9] Bergström Anna, Hulchiy Olesya, Kull Inger, Lind Tomas, Melén Erik, Moskalenko Vitaliy, Pershagen Göran, Bellander Tom, Gruzieva (2013). Exposure to air pollution from traffic and childhood asthma until 12 years of age. Epidemiology.

[10] Breysse PN, Diette GB, Matsui EC, Butz AM, Hansel NN, McCormack MC (2010). Indoor Air Pollution and Asthma in Children. Proceedings of the American Thoracic Society.

[11] Franklin PJ (2007). Indoor air quality and respiratory health of children. Paediatric Respiratory Reviews.

[12] Akar-Ghibril N, Phipatanakul W (2020). The Indoor Environment and Childhood Asthma. Curr Allergy Asthma Rep.

[13] Martinez (2009). Managing childhood asthma: challenge of preventing exacerbations. Pediatrics.

[14] Guo H, Huang S, Chen M (2018). Air pollutants and asthma patient visits: Indication of source influence. Science of The Total Environment.

[15] Garcia E, Berhane KT, Islam T, McConnell R, Urman R, Chen Z, Gilliland FD (2019). Association of changes in air quality with incident asthma in children in California, 1993-2014. JAMA.

[16] Anderson K, Burford O, Emmerton L (2016). App chronic disease checklist: protocol to evaluate mobile apps for chronic disease self-management. JMIR Res Protoc.

[17] Ranjan Y, Rashid Z, Stewart C, Conde P, Begale M, Verbeeck D, Boettcher S, Dobson R, Folarin A (2019). RADAR-Base: open source mobile health platform for collecting, monitoring, and analyzing data using. JMIR Mhealth Uhealth.

[18] Camacho-Rivera M, Vo H, Huang X, Lau J, Lawal A, Kawaguchi A (2020). Evaluating asthma mobile apps to improve asthma self-management: user ratings and sentiment analysis of publicly available apps. JMIR Mhealth Uhealth.

[19] Ramsey RR, Plevinsky JM, Kollin SR, Gibler RC, Guilbert TW, Hommel KA (2020). Systematic review of digital interventions for pediatric asthma management. The Journal of Allergy and Clinical Immunology: In Practice.

[20] Tinschert P, Jakob R, Barata F, Kramer J, Kowatsch T (2017). The potential of mobile apps for improving asthma self-management: a review of publicly available and well-adopted asthma apps. JMIR Mhealth Uhealth.

[21] Kim S, Park Y, Ackerman MK (2021). Designing an indoor air quality monitoring app for asthma management in children: user-centered design approach. JMIR Form Res.

[22] Airnow.

[23] National Heart and Lung and Blood Institute. National Asthma Education Program. Expert Panel on the Management of Asthma (1991). Guidelines for the diagnosis and management of asthma. National Asthma Education Program, Office of Prevention, Education.

[24] Markopoulos P, Bekker M (2003). Interaction design and children. Interacting with Computers.

[25] Braun V, Clarke V (2006). Using thematic analysis in psychology. Qualitative Research in Psychology.

[26] Chadia Abras, Maloney-Krichmar Diane, Jenny Preece (2004). User-centered design. Bainbridge, W. Encyclopedia of Human-Computer Interaction. Thousand Oaksage Publications 37, no. 4 (2004).

[27] O'Brien HL, Toms EG (2008). What is user engagement? A conceptual framework for defining user engagement with technology. J. Am. Soc. Inf. Sci.

[28] Sunyoung Kim, Eric Paulos, Jennifer Mankoff (2013). inAir: a longitudinal study of indoor air quality measurements and visualizations.

[29] Tang J, Abraham C, Stamp E, Greaves C (2014). How can weight-loss app designers' best engage and support users? A qualitative investigation. Br J Health Psychol.

[30] Bhatia A, Kara J, Janmohamed T, Prabhu A, Lebovic G, Katz J, Clarke H (2021). User engagement and clinical impact of the manage my pain app in patients with chronic pain: a real-world, multi-site trial. JMIR Mhealth Uhealth.

[31] Rúben Gouveia, Evangelos Karapanos, Marc Hassenzahl (2015). How do we engage with activity trackers? A longitudinal study of Habito.

[32] Petersen JM, Prichard I, Kemps E (2019). A comparison of physical activity mobile apps with and without existing web-based social networking platforms: systematic review. J Med Internet Res.

[33] Böhm A, Jensen ML, Sørensen MR, Stargardt T (2020). Real-world evidence of user engagement with mobile health for diabetes management: longitudinal observational study. JMIR Mhealth Uhealth.

[34] Turchioe MR, Heitkemper EM, Lor M, Burgermaster M, Mamykina L (2019). Designing for engagement with self-monitoring: A user-centered approach with low-income, Latino adults with Type 2 Diabetes. International Journal of Medical Informatics.

[35] Miyamoto SW, Henderson S, Young HM, Pande A, Han JJ (2016). Tracking health data is not enough: a qualitative exploration of the role of healthcare partnerships and mhealth technology to promote physical activity and to sustain behavior change. JMIR mHealth uHealth.

[36] Christina N, Harrington (1111). , Ljilja Ruzic,Jon A.

[37] Hightow-Weidman L, Muessig K, Knudtson K, Srivatsa M, Lawrence E, LeGrand S, Hotten A, Hosek S (2018). A gamified smartphone app to support engagement in care and medication adherence for HIV-positive young men who have sex with men (AllyQuest): development and pilot study. JMIR Public Health Surveill.

[38] Gabe Zichermann, Christopher Cunningham (2011). Gamification by design: implementing game mechanics in web and mobile apps. O'Reilly Media, Inc.

[39] Miller AS, Cafazzo JA, Seto E (2014). A game plan: Gamification design principles in mHealth applications for chronic disease management. Health Informatics J.

[40] Habre R, Coull B, Moshier E, Godbold J, Grunin A, Nath A, Castro W, Schachter N, Rohr A, Kattan M, Spengler J, Koutrakis P (2013). Sources of indoor air pollution in New York City residences of asthmatic children. J Expo Sci Environ Epidemiol.

[41] Leung DYC (2015). Outdoor-indoor air pollution in urban environment: challenges and opportunity. Front. Environ. Sci.

[42] David DavidShaffer, James Paul Gee (2006). How computer games help children learn.

[43] Habgood MPJ, Ainsworth SE (2011). Motivating children to learn effectively: exploring the value of intrinsic integration in educational games. Journal of the Learning Sciences.

[44] Lissak G (2018). Adverse physiological and psychological effects of screen time on children and adolescents: Literature review and case study. Environmental Research.

